# Sinorhizobium meliloti, a Slow-Growing Bacterium, Exhibits Growth Rate Dependence of Cell Size under Nutrient Limitation

**DOI:** 10.1128/mSphere.00567-18

**Published:** 2018-11-07

**Authors:** Xiongfeng Dai, Zichu Shen, Yiheng Wang, Manlu Zhu

**Affiliations:** a School of Life Sciences, Central China Normal University, Wuhan, China; University of Iowa

**Keywords:** *Sinorhizobium meliloti*, cell cycle, cell size, growth rate

## Abstract

Bacterial cells need to coordinate the cell cycle with biomass growth to maintain cell size homeostasis. For fast-growing bacterial species like Escherichia coli and Bacillus subtilis, it is well-known that cell size exhibits a strong dependence on the growth rate under different nutrient conditions (known as the nutrient growth law). However, cell size changes little with slow growth (doubling time of >90 min) for E. coli, posing the interesting question of whether slow-growing bacteria species also observe the nutrient growth law. Here, we quantitatively characterize the cell size and cell cycle parameter of a slow-growing bacterium, Sinorhizobium meliloti, at different nutrient conditions. We find that S. meliloti exhibits a threefold change in its cell size when its doubling time varies from 2 h to 6 h. Moreover, the progression rate of its cell cycle is much longer than that of E. coli, suggesting a delicate coordination between the cell cycle progression rate and the biomass growth rate. Our study shows that the nutrient growth law holds robustly regardless of the growth capacity of the bacterial species, generalizing its applicability among the bacterial kingdom.

**IMPORTANCE** The dependence of cell size on growth rate is a fundamental principle in the field of bacterial cell size regulation. Previous studies of cell size regulation mainly focus on fast-growing bacterial species such as Escherichia coli and Bacillus subtilis. We find here that Sinorhizobium meliloti, a slow-growing bacterium, exhibits a remarkable growth rate-dependent cell size pattern under nutrient limitation, generalizing the applicability of the empirical nutrient growth law of cell size. Moreover, S. meliloti exhibits a much slower speed of cell cycle progression than E. coli does, suggesting a delicate coordination between the cell cycle progression rate and the biomass growth rate.

## Abstract (precis)

The dependence of cell size on growth rate is a fundamental principle in the field of bacterial cell size regulation. Previous studies of cell size regulation mainly focus on fast-growing bacterial species such as Escherichia coli and Bacillus subtilis. We find here that Sinorhizobium meliloti, a slow-growing bacterium, exhibits a remarkable growth rate-dependent cell size pattern under nutrient limitation, generalizing the applicability of the empirical nutrient growth law of cell size. Moreover, S. meliloti exhibits a much slower speed of cell cycle progression than E. coli does, suggesting a delicate coordination between the cell cycle progression rate and the biomass growth rate.

## OBSERVATION

Understanding how cells maintain size homeostasis remains a grand challenge in biology (1–3). Bacterial cells manage to coordinate biomass growth with cell cycle progression, including chromosome replication and cell division to maintain size homeostasis (3–5). The growth of biomass can exert a profound effect on the cell size of bacteria, as indicated by the well-known positive dependence of cell size on growth rate for Escherichia coli and Bacillus subtilis under different nutrient conditions (known as the nutrient growth law) (1, 3, 6–9). The growth rate of E. coli and B. subtilis can be easily altered from 20 min per doubling to several hours per doubling by supplying different nutrient sources (1, 6, 7, 10). However, there also exist many slow-growing species with their shortest generation times being several hours in the bacterial kingdom (11). It remains unclear whether nutrient limitation could also lead to a similar growth-dependent cell size pattern in slow-growing bacterial species. Moreover, little is known about the effect of growth rate on cell cycle and DNA content in slow-growing bacterial species.

Sinorhizobium meliloti is a slow-growing bacterium that is capable of conducting symbiotic nitrogen fixation upon interacting with its legume host, the *Medicago* plant (12, 13). During the process of symbiotic nitrogen fixation, the host plant generates a family of small host peptides called nodule-specific cysteine-rich (NCR) peptides to modulate the cell cycle progression of S. meliloti, further stimulating the conversion of bacterial cells into bacteroids, which are much larger than free-living cells (12, 14, 15). The above process is a key step for the success of symbiotic nitrogen fixation. Therefore, it is naturally interesting to investigate the cell size and cell cycle of S. meliloti due to its crucial role in nitrogen fixation. In this study, we quantitatively investigate the cell size and cell cycle progression of free-living S. meliloti cells growing under different nutrient conditions. We found that the positive growth dependence of cell size under nutrient limitation holds well for S. meliloti, generalizing the applicability of the nutrient growth law among the bacterial kingdom.

**FIG 1:**
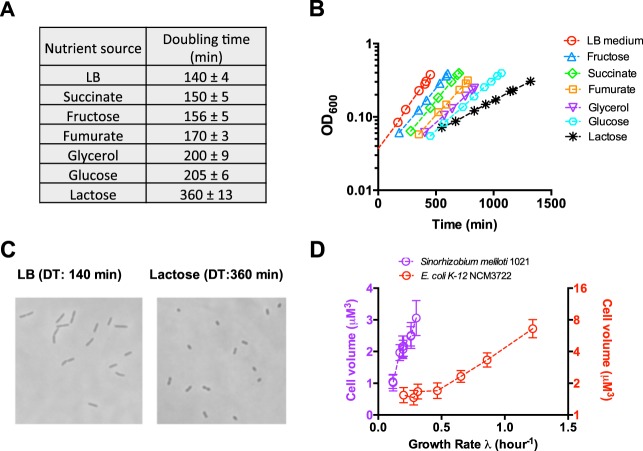
Dependence of cell size of Sinorhizobium meliloti 1021 on growth rate under nutrient limitation. (A) The doubling time (DT) of S. meliloti growing in different nutrient conditions at 30°C. (B) Exponential growth curves of S. meliloti growing in different nutrient conditions. (C) Images of S. meliloti cells in LB medium and minimal medium containing lactose. (D) Quantitative correlation between the cell size and growth rate for both S. meliloti and E. coli at 30°C. Nutrient conditions used for E. coli include LB medium (DT of 34 min), medium containing Casamino acid and glucose (48 min), medium containing glucose (63 min), medium containing glycerol (90 min), medium containing acetate (135 min), medium containing mannose (150 min), and medium containing aspartate (210 min). Data are averages for triplicates with standard deviations being within 10%.

We focus on S. meliloti 1021 strain growing exponentially under different nutrient conditions at 30°C. By varying the carbon sources in the minimal medium, the growth rate could be altered from 150 min (succinate-containing medium) per doubling to 360 min (lactose-containing medium) per doubling (Fig. 1A and B). For cells growing in rich Luria-Bertani (LB) medium, the growth rate (140 min per doubling) is only a bit faster than that of cells growing in succinate-containing medium (150 min per doubling). Therefore, the growth capacity of S. meliloti is much lower than that of E. coli and B. subtilis. Images of the cells in exponentially growing cultures under each condition were taken by phase-contrast microscopy to analyze the cell size. Remarkably, the cell size of S. meliloti decreases dramatically by 70% for bacteria grown on LB medium to lactose-containing medium (Fig. 1C). Therefore, S. meliloti displays a strong growth-dependent cell size pattern under nutrient limitation. For comparison, we also characterized the cell size of E. coli K-12 cells growing under different nutrient conditions at 30°C (9, 16). As shown in Fig. 1D, the cell size of E. coli also decreases strongly at lower growth rate, which had been well-known (6, 9). However, at a slow growth rate (λ < 0.4 h^−1^), the cell size of E. coli changes little, while the cell size of S. meliloti varies threefold (Fig. 1D). Overall, the above finding demonstrates that the slow-growing S. meliloti exhibits an even stronger growth-dependent cell size pattern than E. coli does under nutrient limitation.

**FIG 2:**
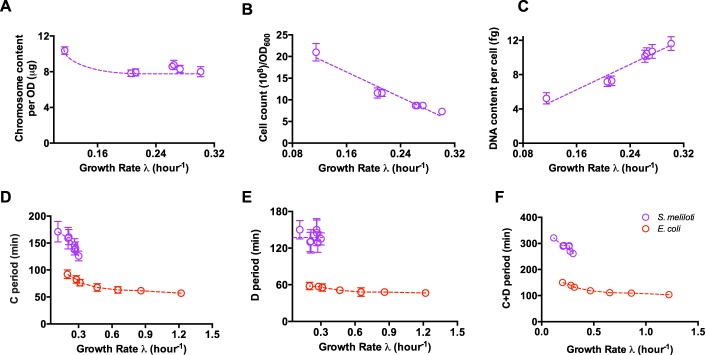
Dependence of chromosome content and cell cycle parameters of Sinorhizobium meliloti 1021 on growth rate. (A) Total chromosome content per mass; (B) cell count per OD_600_; (C) DNA content per cell; (D) C period of S. meliloti and E. coli. The E. coli data were measured at a temperature below 30°C. (E) D period of S. meliloti and E. coli. The E. coli data were measured at a temperature below 30°C. (F) C period plus D period of S. meliloti and E. coli. Data are averages for triplicates with standard deviations being within 10%.

Since cell size homeostasis is tightly associated with the cell cycle, we next characterized the cellular DNA content and cell cycle parameters of S. meliloti. Total DNA content per mass (optical density at 600 nm [OD_600_]) increases only slightly with decreasing growth rate (Fig. 2A). The number of S. meliloti cells increases by threefold with decreasing growth rate (Fig. 2B), following the opposite trend of cell size and confirming that the product of cell size and cell number is a good proxy of total cell mass (OD_600_). On the basis of the cell number result, we found that the average DNA content per cell of S. meliloti was also positively correlated with the growth rate under nutrient limitation (Fig. 2C), similar to the finding with E. coli (9). Moreover, fast-growing cells are 2.5 times the DNA content of slow-growing cells, suggesting the existence of multireplication forks.

The bacterial cell cycle contains two key stages, the C period and D period (3–5, 17). The C period refers to the time required for chromosome replication. The D period refers to the time between the completion of chromosome replication and cell division. Strikingly, the C period of S. meliloti increases from ∼2 h to ∼3 h under nutrient limitation (Fig. 2D). This value is much higher than that of E. coli at 30°C, which is ∼50 min, indicating that the movement speed of the DNA replication fork in S. meliloti is much slower than that in E. coli. Similarly, the D period of S. meliloti is also much longer than that of E. coli, suggesting a much slower cell division process as well (Fig. 2E). Overall, the above finding demonstrates that S. meliloti has a much slower cell cycle progression rate than E. coli does (Fig. 2F).

In conclusion, our findings show that the positive growth rate-dependent cell size and cellular DNA content under nutrient limitation also hold for the slow-growing S. meliloti. These findings support the general applicability of the nutrient growth law regardless of the bacterial growth capacity. Moreover, S. meliloti has a much slower cell cycle progression rate than E. coli does, suggesting an attractive coordination between the cell cycle progression rate and biomass growth rate. In the future, it will be fascinating to investigate the molecular basis of the nutrient growth law of S. meliloti as well as the intrinsic limiting factors of cell cycle progression among different bacterial species.

### Strains and medium.

The strains used in this study are either wild-type E. coli K-12 NCM3722 (16) or Sinorhizobium meliloti 1021 (14). E. coli was grown on MOPS-buffered minimal medium supplemented with different carbon sources or nitrogen sources (9, 16). S. meliloti was grown on M9 minimal medium supplemented with different carbon sources (18, 19).

### Growth rate measurement.

Cell growth is performed in a 30°C water bath shaker (220 rpm). The cell growth procedure contains three steps: seed culture, preculture, and experimental culture. For seed culture, cells in a fresh LB agar plate were inoculated into LB broth and grown for several hours. For preculture, the seed cultures were then transferred to the medium of the experimental culture (e.g., minimal medium containing glucose) and grown overnight at 30°C. For experimental culture, on the next day, the overnight precultures were inoculated into the same medium as the medium used for preculture at an initial OD_600_ of ∼0.03. For each condition, 6 to 8 OD_600_ data points (ranging from OD_600_ values of 0.05 to 0.5) were taken to obtain an exponential growth curve for calculating the growth rate. The OD_600_ values were measured by a Thermo Sci Genesys 30 spectrophotometer.

### Cell size measurement.

Five to 10 μl of cell culture at an OD_600_ of ∼0.3 was added to a slide glass covered with a thin layer of agar to immobilize the cells. Phase-contrast cell images were taken using a Nikon Eclipse Ti-80 microscope. For each condition, the images of 500 to 1,000 individual cells were taken for size analysis. Cell length (L) and width (W) of each cell were taken using the ImageJ software. The cell volume (V) was calculated based on $V=\pi W^{2}/4(L-\frac{W}{3}).$

### DNA content and cell cycle measurement.

The C period was measured by the DNA increment method as described by Churchward et al. (20) and Bipatnath et al. (21). This method is based on measuring the DNA increment after blocking DNA initiation of exponentially growing cells by the addition of chloramphenicol (300 µg/ml) or rifampin (200 µg/ml) (runoff experiments). For S. meliloti cells, we used 0.3% (vol/vol) 2-phenethanol to block the DNA replication initiation process (22).

The total DNA content per OD_600_ was measured by the diphenylamine colorimetric method as detailed by Basan et al. (9). DNA content per cell was obtained by measuring the total amount of DNA per OD and cell number per OD by plating or by using a bacterial counting chamber and microscopy. The D period is obtained with the C-period data and DNA content data as described by Si et al. (4).
